# Psychometric properties of the Feedback Orientation Scale in the clinical workplace of health professions students

**DOI:** 10.5116/ijme.679e.07de

**Published:** 2025-02-25

**Authors:** Javiera Fuentes-Cimma, Dominique Sluijsmans, Paulina Perez-Mejias, Ignacio Villagran, Arnoldo Riquelme, Sylvia Heeneman

**Affiliations:** 1 School of Health Sciences, Faculty of Medicine, Pontificia Universidad Católica de Chile, Santiago, Chile; 2 Rotterdam University of Applied Sciences, Rotterdam, the Netherlands; 3 School of Medicine, Johns Hopkins University, Baltimore, United States of America; 4 Centre for Medical and Health Profession Education, Faculty of Medicine, Pontificia Universidad Católica de Chile, Santia-go, Chile; 5 Department of Pathology, Faculty of Health, Medicine and Health Sciences, Maastricht University, the Netherlands

**Keywords:** Feedback, feedback orientation, health professions education, clinical workplace, self-efficacy

## Abstract

**Objectives:**

To cross-culturally validate the Feedback Orientation Scale in the clinical workplace, focusing on the Spanish adaptation of the instrument in the Chilean context.

**Methods:**

A cross-cultural validation of the Feedback Orientation Scale was conducted across six Chilean universities and nine health professions education programs. The target population were students in their clinical clerkship. The scale was translated through a rigorous process and was applied online. Validity and reliability of the constructs were evaluated through confirmatory factor analysis. A descriptive statistical analysis was conducted.

**Results:**

A total of 510 students participated (70% female, average age 24.1 years, 30% response rate). Students' responses were from Medicine (n=128), Physiotherapy (n=128), Nursing (n=63), Dentistry (n=49), and five other disciplines. Confirmatory factor analysis showed a sufficient fit of the original factor structure CFI = 0.96, SRMR = 0.045, RMSEA = 0.051, 90% CI [0.044, 0.057]. Item loadings were above 0.50. Factor reliability ranged from 0.77 to 0.91. Overall, students’ perception of receptivity to feedback was positive, and the Feedback Self-efficacy subscale had the most "disagree" and "strongly disagree" responses.

**Conclusions:**

Our findings provide evidence regarding the validity and reliability of the Feedback Orientation Scale for assessing the feedback orientation of health profession education students in the clinical workplace. Students scored lowest on two items related to feedback self-efficacy, indicating low confidence in handling feedback. This Feedback Orientation Scale can reveal valuable insights into how students may differ in their receptivity and use of feedback in the clinical workplace, informing teaching practices and interventions, and redesigning existing feedback practices.

## Introduction

The new paradigms in feedback emphasize a shift from focusing solely on feedback delivery towards interaction, sense-making, and the active engagement of students in their feedback processes.^1^^,^^2^ Feedback orientation is a multidimensional construct defined as a person’s overall receptivity to feedback.^3^^,^^4^ It is influenced by an individual´s sensitivity to others' views or opinions and their perceived accountability to act upon information that is considered credible. Feedback orientation helps to understand individual differences in reaction to feedback, how feedback is interpreted, and how feedback receivers use feedback.^3^ Consequently, examining students' feedback orientation can enhance our understanding of how they perceive and use feedback within the clinical educational context.

A recognized instrument for measuring feedback orientation is the Feedback Orientation Scale (FOS). It was initially designed for use in the non-clinical organizational workplace to evaluate the individual characteristics that influence feedback orientation.^5^ The FOS has demonstrated successful validation across various contexts and cultural settings. Kartol and Arslan^6^ reported evidence for FOS validity to the Turkish culture to be applied to university teacher students and Lilford and colleagues.^7^ to the South African culture to assess feedback orientation in salespersons. Braddy and colleagues.^8^ used the FOS to evaluate the feedback orientation of middle-to-senior leaders, and Mrazek^9^ surveyed employees of three different generations (i.e., for-profit, not-for-profit, and higher education). Dahling and colleagues.^10^ applied the FOS to employed teaching students, showing that feedback orientation was positively related to job performance and feedback-seeking behavior. Regarding health-related contexts, Imanipour and colleagues.^11^ applied this survey to medical and nursing students after adapting it to Persian without reporting psychometric properties or details of the context adaptation. Recently, Mills and colleagues.^12^ used the FOS for a cross-sectional analysis of feedback orientation in medical students and internal medicine residents at one large academic center and found equivalence of the same dimensions to feedback orientation as in management sciences.

Surveys should provide comparable and generalizable results across cultures or languages.^13^ As health professionals rely on cultural and linguistic understanding when conducting surveys, conducting cross-cultural validation studies of instruments is imperative.^14^ In the case of the FOS, the authors of the original publication called for its adaptation to other contexts and samples.^5^ Given the importance of understanding feedback orientation during clinical learning of health profession students, this study aimed to cross-culturally validate the FOS in the clinical workplace in an undergraduate setting, specifically focusing on the Spanish adaptation of the instrument in the Chilean context. It was studied whether the original FOS questionnaire constructs were equivalent in the clinical workplace.

## Methods

### Study design and participants

The design was a cross-cultural validation study conducted following the steps proposed by Beaton and colleagues.^13^ and Sousa and Rojjanasrirat.^14^ Eligible survey participants included students enrolled in health professional programs and taking clerkship-level courses at six Chilean universities (n=1,750). A sample size of at least 400 responses was estimated because the fit indices show their asymptotic properties from this number onwards.^15^ Non-probability convenience sampling was used.

Ethical approval was obtained from the Social and Humanities Ethics Committee of the Pontificia Universidad Católica de Chile.

### Data collection

The FOS is a self-reported 20-item survey scored on a 5-point Likert scale (i.e., 1=strongly disagree to 5=strongly agree). It comprises four subscales: Utility, Accountability, Social awareness, and Feedback self-efficacy. The Utility subscale is related to the extent to which an individual believes that using feedback results in beneficial outcomes. The Accountability subscale relates to the sense of self-responsibility in acting upon the feedback information; the Feedback self-efficacy subscale reflects an individual´s confidence in dealing with feedback; and the social awareness subscale encompasses the individual´s tendency to be aware of other’s views of themselves using feedback and to be sensitive to these views.^5^

We conducted forward-and-back-translated versions of the instrument to adapt the FOS in a different educational context and language.^13^^,^^14^ First, the FOS underwent a forward translation process from English to Spanish conducted by two certified interpreters who were bilingual (i.e., fluent in English and Spanish) and bicultural (i.e., experienced living in an Anglo speaker and Chilean cultures), resulting in two forward-translated versions. A third bilingual team member compared these versions with the original and resolved any ambiguities or discrepancies through discussion involving two research team members, the interpreters, and the main investigator. This step led to the creation of a preliminary initial translated version of the FOS. Then, back translation was conducted by two bilingual interpreters who performed a blind-back translation of the preliminary initial version back to the original language. This produced two back-translated versions of the FOS. A committee comprising three team members and the interpreters compared these versions with each other and with the original instrument. This step resulted in a pre-final version of the instrument in the original language.

To ensure the clarity and understanding of the Spanish version, a pilot study was conducted with a group of students from one university. They provided feedback on their comprehension of the instructions and items and rated the clarity of each item. The pre-final version of the translated instrument underwent full psychometric testing.

### Procedure

Invitations to participate in the survey were sent to students in the Spring of 2021 during the COVID-19 pandemic. Students were informed that participation was voluntary and that their responses would be anonymous. A survey link was sent to each student via an online survey platform, and the survey remained open for six weeks, with reminders sent every two weeks.

Data collected included gender, age, enrolment year, and program.

### Data analysis

We used confirmatory factor analysis (CFA) to assess the reliability and factorial validity of the FOS scale. Factor reliability was assessed using the McDonald’s ω, a coefficient that measures the internal consistency among indicators loading on the same factor. This coefficient is defined as the proportion of the total variance of each factor that can be attributed to the common variance across indicators.^16^ Reliability estimates above 0.7 are generally considered acceptable. We chose McDonald’s ω instead of Cronbach’s α because the latter assumes all items have the same influence on the underlying factor (i.e., equal factor loadings). This assumption is not met by the original FOS scale. In contrast, McDonald's omega is more appropriate when the factor loadings and the error variance vary across items.^17^

The CFA framework not only assesses factor reliability but also provides valuable insights into individual reliability at the item level. Specifically, item reliability can be assessed by looking at the R-squared coefficients from the standardized, which indicate the proportion of variance in an indicator explained by the underlying factor. R-square values exceeding 0.5 are generally considered acceptable, suggesting the item is reliably measuring the intended construct.^18^

In a standardized solution, factor loadings can be interpreted as regression coefficients, i.e., one standard deviation increase in the factor is associated with the coefficient value increase in the indicator. Therefore, the higher the factor loading, the better the association between the factor and the respective indicator. Careful examination of factor loading magnitudes is crucial in assessing whether each indicator adequately represents its underlying latent construct. In practice, completely standardized factor loadings of 0.3 or 0.4 and above are often considered the threshold for factor loadings to be considered "salient".^18^

Finally, another important piece of information provided by CFA models is the correlation among factors, which indicates the discriminant validity of the latent constructs. A factor correlation exceeding 0.80 or 0.85 is considered indicative of poor discriminant validity, i.e., factors may not represent distinct constructs.^18^

### Model Specification

We fitted three alternative models that fit the same factorial structure specified for the original FOS instrument: (1) a one-factor model with all items loading on one factor, (2) a first-order factor model in which items were allowed to load on four factors corresponding to each FOS construct, and (3) a second-order model with items loading on four first-order factors, and each of them loading on one second-order factor.

We used Mplus 8.7 to estimate the models. We initially specified each alternative model, fixing all error covariances to zero and allowing free estimation of factor loadings, variances, and error variances. Subsequently, we changed these initial settings by incorporating error covariances between observed indicators loading on the same factors guided by the model modification indices provided by Mplus.

We assessed the CFA model's fit using the Comparative Fit Index (CFI), Root Mean Square Error of Approximation (RMSEA), and Standardized Root Mean Square Residual (SRMR). We used Hu and Bentler's (1999) cutoff benchmarks, with values of CFI ≥ 0.96, RMSEA ≤ 0.06, and SRMR ≤ 0.09, suggesting a good model fit.^19^

## Results

The FOS was adapted to the clinical workplace following the aforementioned steps. In addition, cognitive debriefing with a group of clinical teachers and students was performed to make minor adaptations of some items to fit the clinical workplace context. For example, in item 4, the original survey stated, "Feedback from supervisors can help me advance in a company, " in the present version, this item states, "Feedback from my supervisors can help me progress in my rotation/workplace".

In total, 510 students responded to the survey (70% (n=392) were female), with an average age of 24.1 years (SD=2.1) and an average response rate of 30%. Table 1 shows the distribution of students according to gender, age, enrolment year, and program of the participants.

**Table 1:** Demographic characteristics of the sample

Demographic Categories		N	%
Gender			
	Female	357	70
	Male	150	29
	Other	3	1
Age			
	Less than 23 years old	83	16
	23	116	23
	24	132	26
	25	81	16
	26	44	9
	More than 26 years old	54	11
Year of program entry			
	2014 or before	42	8
	2015	102	20
	2016	174	34
	2017	192	37
Program			
	Physiotherapy	128	25
	Medicine	128	25
	Nursing	63	12
	Dentistry	49	10
	Nutrition	27	5
	Speech Pathology	38	7
	Medical Technology	29	6
	Midwifery	9	2
	Occupational Therapy	39	8

**Figure 1:**
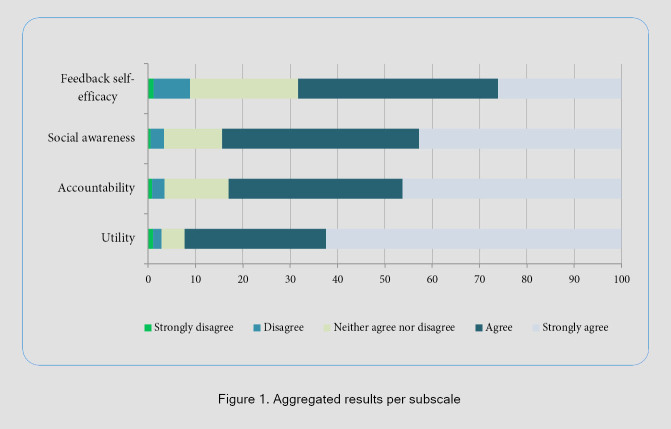
Aggregated results per subscale

Overall, the responses showed that the student’s perception of receptivity to feedback was positive, with a high percentage of "agree" and "strongly agree" responses in the Utility and social awareness subscale. The highest-rated items were both from the Utility subscale: "*Feedback from supervisors can help me progress in my rotation/workplace*" and "*Feedback contributes to my success in clinical work*," with 95.5% (n=487) and 94.1% (n=480) of "agree" or "strongly agree" responses respectively. The subscale Feedback Self-efficacy showed the lowest rated items: *"I feel confident when responding to both positive and negative feedback."* and *"Compared to others, I am more competent at dealing with feedback”,* with 15.5% (n=80) and 10.6% (n=54) "disagree" or "strongly disagree" responses, respectively. The aggregated results per subscale showed that the subscale Feedback Self-efficacy is the one with the highest proportion of "disagree" and "strongly disagree" responses compared to the other subscales (Figure 1).

**Table 2:** Fit indices for the three estimated models using confirmatory factors analysis

Model	c^2^	df	SRMS	RMSEA	CFI
One-factor model	2870.684	170	0.139	0.176 [CI 90%, 0.171, 0.182]	0.517
First-order model	366.871	159	0.045	0.051 [CI 90%, 0.044, 0.057]	0.963
Second-order model	376.732	162	0.053	0.051 [CI 90%, 0.044, 0.058]	0.962
Acceptable threshold^19^	-	-	≤ 0.09	≤ 0.06	≥ 0.96

**Table 3:** First-order model parameter estimates (completely standardized solution)

Statements		Factor Loadings		R-Square		Factor Reliability
		Estimate	SE	Estimate	SE	
Utility						0.909
	Feedback contributes to my success at work.	0.793	0.020	0.628	0.032	
	I rely on feedback to improve my skills at work.	0.824	0.018	0.679	0.029	
	Feedback is critical to improve my performance.	0.842	0.016	0.709	0.027	
	Feedback from my supervisors can help me progress in my rotation/workplace.	0.837	0.017	0.701	0.028	
	I believe that feedback is essential to reach my goals.	0.791	0.020	0.625	0.031	
Accountability						0.773
	It is my responsibility to apply feedback to improve my performance.	0.564	0.039	0.318	0.044	
	I feel accountable for responding to feedback appropriately.	0.758	0.039	0.574	0.044	
	I do not feel a sense of closure until I respond to feedback.	0.583	0.036	0.339	0.042	
	If my supervisor gives me feedback, I have the responsibility to act on it.	0.709	0.031	0.503	0.044	
	I feel obligated to make changes based on the feedback I receive.	0.615	0.037	0.378	0.045	
Social Awareness						0.871
	I try to be aware of what other people think of me.	0.570	0.033	0.324	0.038	
	Feedback makes me more aware of what other people think of me.	0.719	0.025	0.516	0.036	
	Feedback helps me manage the impression I make on others.	0.865	0.016	0.748	0.028	
	Feedback lets me know how I am perceived by others.	0.842	0.018	0.709	0.030	
	I rely on feedback to help me make a good impression.	0.736	0.024	0.542	0.035	
Feedback Self-Efficacy						0.866
	I feel self-assured when dealing with feedback.	0.726	0.025	0.527	0.037	
	Compared to others, I am more competent at dealing with feedback.	0.670	0.028	0.449	0.038	
	I believe I can deal with feedback effectively.	0.855	0.018	0.732	0.031	
	I feel confident when responding to both positive and negative feedback.	0.763	0.024	0.582	0.037	
	I know I can handle the feedback I receive.	0.766	0.023	0.587	0.036	
Factor Correlations						-
	Utility – Accountability	0.637	0.037	-	-	
	Utility – Social Awareness	0.407	0.042	-	-	
	Accountability – Social Awareness	0.549	0.042	-	-	
	Utility – Feedback Self-Efficacy	0.353	0.045	-	-	
	Accountability – Feedback Self-Efficacy	0.408	0.048	-	-	
	Social Awareness – Feedback Self-Efficacy	0.225	0.048	-	-	

**Figure 2:**
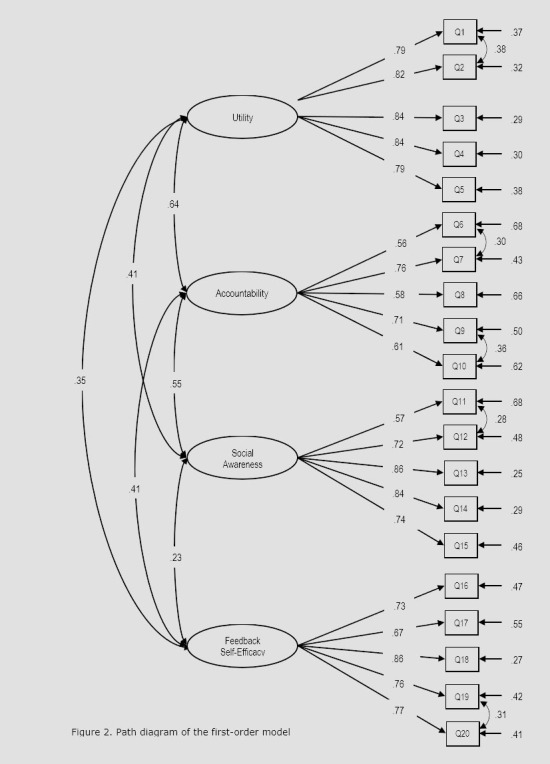
Path diagram of the first-order model

CFA was used to test the factorial validity and reliability of the instrument. The absolute, relative, and parsimony fit indices of the three alternative estimated models are provided in Table 2. The one-factor model displays poor fit indices, while the first- and second-order models both fit the data well. However, the first-order model has a slightly better SRMR fit, indicating that the average magnitude of the discrepancies between observed and expected correlations is slightly smaller for the first-order model. Therefore, this model is preferred. The excellent fit of the first-order model is evidence of the factorial validity of the FOS. Figure 2 represents the path diagram of the first-order model, where each FOS construct, represented by a circle, is a latent factor being measured by its corresponding set of observed indicators, represented by squares. Paths connecting indicators and factors represent factor loadings, while paths connecting factors represent factor correlations.

The estimates for factor reliabilities all fall above the desired threshold of 0.7 (Table 3). The estimates of factor reliability indicate that 91%, 77%, 87%, and 87% total variance of the factors Utility, Accountability, Social Awareness, and Feedback Self-efficacy can be attributed to the common variance of their respective indicators, respectively. This means that the factors are reliable measures of the intended underlying construct. Also, all factor correlation estimates are positive and fall below 0.8. This is evidence of strong construct discriminant validity, meaning that all factors are valid measures of distinct FOS underlying constructs.

As for the size of factor loadings, all estimates have values above 0.4, so they all can be deemed salient factor loadings. However, items 6, 8, 10, and 11 display R-square coefficient estimates below the 0.5 desired cutoff value. This means these items may not be reliable measures of their corresponding factors.

## Discussion

The aim of this study was to cross-culturally validate the FOS in the undergraduate clinical workplace, specifically focusing on the Spanish adaptation of the instrument in the Chilean context, which was achieved through a rigorous cross-cultural adaptation.^13^^,^^14^ The present study showed that the FOS could be adapted to the clinical workplace, a challenging learning context for students and instructors. Evidence was provided regarding the validity and reliability of the adapted FOS for assessing the feedback orientation of health profession students in the clinical workplace. The findings showed that the FOS can provide insights to evaluate differences that could make students more receptive to feedback in this challenging context.

In response to the authors' call to test this scale in other samples and contexts, the measurement properties of the FOS were evaluated in a context and culture different from the original.^5^ To the best of our knowledge, this is the first study exploring the FOS's measurement properties in the (undergraduate) clinical workplace setting. Although two previous studies already used this scale to measure feedback orientation in nursing and medicine students, they did not report evidence of its validity and reliability.^11^^, ^^12^

The confirmatory factor analysis results of our study showed an excellent fit of all items in the subscales as proposed in the original study. The construct validity findings are consistent with the four-factor structure of the original scale5 removing any item after administration to a large sample was unnecessary. Although all factor loadings had satisfactory estimates, items 6, 8, 10, and 11 should be revised or eliminated in subsequent versions of the FOS based on their low R-square coefficients. Regarding factor reliability, the original study reported Cronbach alpha coefficients ranging from 0.74 to 0.86.5 Similarly, our findings determine acceptable reliability on an Omega coefficient ranging from 0.77 to 0.91.

According to the results, the utility subscale yielded the highest scores, while the self-efficacy subscale yielded the lowest. These findings are consistent with a recent study conducted by Mills and colleagues.^12^ Although the literature has not reported a cut-off point for determining when feedback orientation is high or low, in this study, we assumed that overall higher scores describe more feedback-oriented students and lower scores less feedback-oriented students. Even though the original FOS was designed to be used as a whole, each subscale provides interesting insights related to relevant theoretical constructs.^5^ Our findings suggest that these constructs are indeed distinct from one another, based on the discriminant validity evidence (i.e., low to moderate factor correlations). In this regard, the literature reports that high self-efficacy relates to feedback seeking, affecting the opportunities to obtain useful information about their own performance.^20^ Consequently, feedback self-efficacy could impact how feedback information about their performance is delivered and be a starting point for specific coaching strategies.^5^

The two lowest-scoring items were present in the Feedback self-efficacy subscale, indicating that students feel less competent in handling feedback and lack confidence in responding to positive and negative feedback compared to others. These results may be explained by many reasons, one being that students often lack the skills to deal with feedback.^21^

The present study has important implications for practice at different levels. First, at the individual level, understanding differences among students in receiving and using feedback in the clinical workplace can inform teaching practices and interventions to tailor feedback accordingly. For example, a more receptive student may be more available and receptive to specific interventions. Individuals with a strong feedback orientation are more receptive to strategies such as coaching from their supervisors.^3^^, ^^4^^, ^^22^At the same level, the FOS can also provide students with valuable insights into their feedback preferences and processing styles. For example, students with a low feedback orientation might struggle to be receptive to feedback from others. Conversely, students with a strong feedback orientation readily recognize the value of feedback and take ownership of its implementation.

Second, feedback orientation is a construct that can change over time,^3^ so at a program level, the results obtained from the FOS could be used to study cohorts of students regarding their feedback orientation and planning targeted interventions (i.e., coaching, guidance). The organization's workplace literature shows that specific feedback orientation differs across generations, for example, between Generation X, Boomers, and Millenials, so specific feedback strategies are needed to support their receptivity and use of feedback.^9^

Lastly, curriculum designers can use students’ feedback orientation to design aligned feedback opportunities. The ability to use feedback effectively is a critical skill in the learning experience of workplace settings and their relationships.^1^ Consequently, students with strong feedback orientation may naturally engage more readily and effectively in feedback dialogues. Designing feedback processes for these students should focus on empowering their active participation in the learning process. In contrast, groups with weaker feedback orientations might benefit more from creating more structured opportunities for actively interacting with and responding to feedback within the workplace.^5^

### Limitations

This study has limitations. Firstly, although we sent reminders, the response rate was moderate, and the representation across disciplines was uneven. Secondly, the survey was administered online due to the pandemic, which may have affected both the response rate and the student's perception of the feedback process in those uncertain times, leading to a possible bias related to response rate and missing data. Moreover, some participants may lack motivation or interest in completing the survey, leading to incomplete or biased data. For example, participants may have exhibited a social desirability bias, where they provided responses, they believe are socially acceptable, rather than their true opinions or behaviors. Thirdly, our survey was validated in a population of undergraduate health professions students; therefore, our results cannot be extrapolated to postgraduate level or graduated professionals working in clinical settings. Finally, we did not relate feedback orientation to other variables, which would be very interesting in further studies.

## Conclusions

The present results provide evidence of the reliability and validity of the adapted FOS with the original for assessing the feedback orientation of health profession students in the (undergraduate) clinical workplace. Feedback orientation is a multidimensional construct that can impact the education of health professionals at multiple levels. For health professions education, using this scale can reveal valuable insights into how students differ in their receptivity and use of feedback in the clinical workplace. This information can inform teaching practices and interventions, allowing for more tailored and effective feedback. Further research could use the FOS to measure feedback orientation in relation to other variables, such as performance or motivation.

This work was supported with funding from the Health Science Department, Faculty of Medicine, Pontificia Universidad Católica de Chile.

### Conflict of Interest

The author declares that there is no conflict of interest.

## References

[1] Winstone N, Carless D. Designing effective feedback processes in higher education: a learning-focused approach. 1st ed. New York: Routledge; 2020.

[2] Boud D, Molloy E (2013). Rethinking models of feedback for learning: the challenge of design.. Assessment &amp; Evaluation in Higher Education.

[3] London M, Smither JW (2002). Feedback orientation, feedback culture, and the longitudinal performance management process.. Human Resource Management Review.

[4] Gregory JB, Levy PE (2012). Employee feedback orientation: implications for effective coaching relationships.. Coaching: An International Journal of Theory, Research and Practice.

[5] Linderbaum BA, Levy PE (2010). The development and validation of the Feedback Orientation Scale (FOS).. Journal of Management.

[6] Kartol A, Arslan N. Turkish version of the feedback orientation scale: investigation of psychometric properties. Uluslararası Türkçe Edebiyat Kültür Eğitim (TEKE) Dergisi. 2021;10(1):321-9.

[7] Lilford N, Caruana A, Pitt L (2014). Psychometric properties of the feedback orientation scale among South African salespersons.. Psychol Rep.

[8] Braddy PW, Sturm RE, Atwater LE, Smither JW, Fleenor JW (2013). Validating the Feedback Orientation Scale in a leadership development context.. Group &amp; Organization Management.

[9] Mrazek III JC. Investigating and comparing the differences in feedback orientation between three generations [doctoral dissertation]. The University of the Rockies; 2015.

[10] Dahling JJ, Chau SL, O’Malley A (2012). Correlates and consequences of Feedback Orientation in Organizations.. Journal of Management.

[11] Imanipour M, Razaghi Z, Khajeh K (2021). Orientation to receive feedback among medical and nursing students of Shahid Beheshti University of Medical Sciences, 2019.. Journal of Medical Education Development.

[12] Mills LM, O'Sullivan PS, Ten Cate O, Boscardin C (2023). Investigating feedback orientation in medical learners.. Med Teach.

[13] Beaton DE, Bombardier C, Guillemin F, Ferraz MB (2000). Guidelines for the process of cross-cultural adaptation of self-report measures.. Spine (Phila Pa 1976).

[14] Sousa VD, Rojjanasrirat W (2011). Translation, adaptation and validation of instruments or scales for use in cross-cultural health care research: a clear and user-friendly guideline.. J Eval Clin Pract.

[15] Hu LT, Bentler PM, Kano Y (1992). Can test statistics in covariance structure analysis be trusted?. Psychol Bull.

[16] Baldwin SA. Psychological statistics and psychometrics using Stata. 1st ed. Texas: Stata Press; 2019.

[17] McDonald RP. Test theory: a unified treatment. Mahwah, NJ: Lawrence Erlbaum Associates Publishers; 1999.

[18] Brown TA. Confirmatory factor analysis for applied research. 2nd ed. New York, NY: The Guilford Press; 2015.

[19] Hu L, Bentler PM (1999). Cutoff criteria for fit indexes in covariance structure analysis: Conventional criteria versus new alternatives.. Structural Equation Modeling: A Multidisciplinary Journal.

[20] Sherf EN, Morrison EW (2020). I do not need feedback! Or do I? Self-efficacy, perspective taking, and feedback seeking.. J Appl Psychol.

[21] Noble C, Billett S, Armit L, Collier L, Hilder J, Sly C, Molloy E (2020). "It's yours to take": generating learner feedback literacy in the workplace.. Adv Health Sci Educ Theory Pract.

[22] Steelman LA, Wolfeld L (2018). The manager as coach: the role of Feedback Orientation.. J Bus Psychol.

